# Adaptation of avian influenza virus to a swine host

**DOI:** 10.1093/ve/vex007

**Published:** 2017-03-18

**Authors:** Vincent Bourret, Jon Lyall, Simon D W Frost, Angélique Teillaud, Catherine A Smith, Sarah Leclaire, JinQi Fu, Sylvain Gandon, Jean-Luc Guérin, Laurence S Tiley

**Affiliations:** 1 Department of Veterinary Medicine, University of Cambridge, Cambridge, UK; 2 Université de Toulouse, INP, ENVT, Toulouse, France; 3 INRA, UMR 1225, IHAP, Toulouse, France; 4 Centre d’Ecologie Fonctionnelle et Evolutive, UMR CNRS 5175, Montpellier, France

**Keywords:** experimental evolution, virus adaptation, host jump, influenza, epistasis

## Abstract

The emergence of pathogenic RNA viruses into new hosts can have dramatic consequences for both livestock and public health. Here we characterize the viral genetic changes that were observed in a previous study which experimentally adapted a field isolate of duck influenza virus to swine respiratory cells. Both pre-existing and *de novo* mutations were selected during this adaptation. We compare the *in vitro* growth dynamics of the adapted virus with those of the original strain as well as all possible reassortants using reverse genetics. This full factorial design showed that viral gene segments are involved in complex epistatic interactions on virus fitness, including negative and sign epistasis. We also identify two point mutations at positions 67 and 113 of the HA2 subunit of the hemagglutinin protein conferring a fast growth phenotype on the naïve avian virus in swine cells. These HA2 mutations enhance the pH dependent, HA-mediated membrane fusion. A global H1 maximum-likelihood phylogenetic analysis, combined with comprehensive ancestry reconstruction and tests for directional selection, confirmed the field relevance of the mutation at position 113 of HA2. Most notably, this mutation was associated with the establishment of the H1 ‘avian-like’ swine influenza lineage, regarded as the most likely to cause the next influenza pandemic in humans. This multidisciplinary approach to study the genetics of viral adaptation provides unique insights on the underlying processes leading to influenza emergence in a new host species, and identifies specific targets for future surveillance and functional studies.

## 1 Introduction

Influenza A viruses pose grave health threats to humans and animals worldwide. Most influenza A subtypes have been found in wild aquatic birds, which as such provide a global reservoir for these viruses in nature (Slemons et al. 1974; Hinshaw et al. 1982; Fouchier et al. 2005; Obenauer et al. 2006; Olsen et al. 2006). Influenza A viruses commonly exhibit restricted host range, but on occasions can emerge from one species to infect and establish in another. Influenza is thus a threat to humans, pigs, horses, sea mammals, ferrets, mink as well as many terrestrial bird species (Forrest and Webster 2010), and it is believed that many influenza strains circulating in mammals [with the possible exception of bats (Chan et al. 2013; Mehle 2014)] ultimately originated from wild aquatic birds.

Influenza virus is a primary respiratory pathogen of swine, that has direct consequences on pig health, welfare and production (van Reeth 2007; Kuntz-Simon et al. 2010). Influenza infections of swine can also have serious implications for public health. Swine may provide an intermediate host for adaptation of avian influenza strains to humans, which can lead to the generation of viruses with epidemic and pandemic potential. Some swine influenza viruses are reassortants containing various combinations of genes originating from human, avian and swine viruses [reviewed in (Busch et al. 2008)], demonstrating that pigs are susceptible to avian and human viruses. Indeed, phylogenetic trees of influenza genes often show intermingling of swine with avian or human isolates (e.g. Smith et al. 2009a). Pigs are thus seen as a ‘mixing vessel’ for the generation of pandemic influenza viruses through genetic reassortment as well as an adaptive host straddling the avian and human influenza virus gene pools (Scholtissek et al. 1985; Castrucci et al. 1993). This is how the 2009 H1N1 human pandemic (and possibly some of the more deadly human pandemics of the 20th century) ultimately originated. Reconstruction of the cascade of cross-species jumps and reassortments that led to the 2009 pandemic (Smith et al. 2009b) illustrates the pivotal role of swine as an intermediate host. Over 50 documented zoonotic transmission events spanning the years 1958–2005 further stress the frequency of such direct swine-to-human transmissions with associated illness (Myers et al. 2007). Given all that is known regarding its mechanism and potential downstream consequences, the aquatic bird-to-swine host jump is a topic of substantial relevance in influenza epidemiology.

Many strains of avian influenza virus have the capacity to infect swine or swine cells, but most of them do not seem fully adapted to readily spread in swine populations and rather need to evolve further. For instance, in an experimental infection of pigs, titres of an H1N1 avian virus recovered from nasal swabs were lower than those of a swine influenza virus of the same subtype (de Vleeschauwer et al. 2009b). Similar observations were made when comparing swine and duck viruses of different subtypes (Lipatov et al. 2008; de Vleeschauwer et al. 2009a). This suggests that swine-naïve avian viruses are variably and imperfectly adapted to swine hosts. In this context, this study of experimental adaptation focuses on how a duck influenza virus can adapt to more efficient replication in swine respiratory cells.

Duck influenza viruses of the H1 subtype have already adapted to swine with serious consequences (Koçer et al. 2015). Around the beginning of the 20th century, such a jump may have led to the establishment of the ‘classical’ swine influenza lineage (Shope 1931). Uncertainty remains around the timing and precise nature of that event, but a plausible explanation (Gorman et al. 1991) consistent with the phylogenies of contemporary human and swine H1N1 sequences, suggests that cross-transmission from an avian host to humans (possibly after a very transitory adaptation in swine) occurred shortly before 1918. Subsequent transmission from humans to swine in 1918 led to the establishment of the Classical Swine lineage. This lineage dominated until the early 21^st^ century, particularly in the USA but has since dwindled (Vijaykrishna et al. 2011). The best documented of such host jumps however involves a fully avian H1 virus adapting to European pigs in or before 1979 (Pensaert et al. 1981; Brown et al. 1997). This ‘avian-like’ swine lineage went on to become the dominant H1N1 influenza virus in European pigs, as well as spreading widely in Asia. It was the precursor for a number of pathogenic and transmissible strains in pigs and humans, culminating in the 2009 pandemic strain (Smith et al. 2009b), which killed tens of thousands of people in an unforeseen pandemic, and went on to become the dominant seasonal human H1 influenza virus. Perhaps even more importantly, a recent study demonstrated that the ‘avian-like’ swine virus by itself has earned the potential to efficiently transmit among humans and may pose the highest pandemic threat among all influenza viruses currently circulating in animals (Yang et al. 2016).

**Figure 1.**
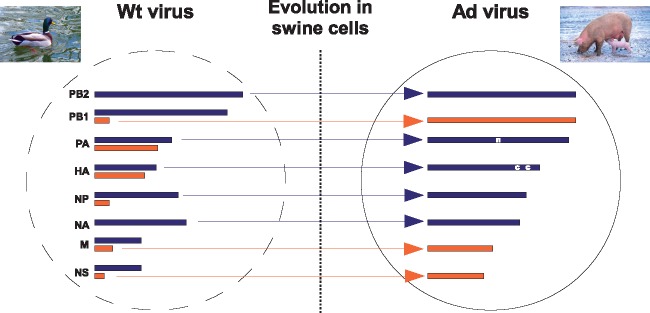
Genetic evolution of the wild type duck isolate following 10 passages in swine cells. The genetic make-up of the wild type (Wt) isolate shows majority gene segments (in blue) co-existing with minority gene segments (in orange). Upon experimental evolution, some minority segments were lost (segments 3, 4, 5, i.e. ‘PA’, ‘HA’, ‘NP’) while others became dominant (segments 2, 7, 8 i.e. ‘PB1’, ‘M’, ‘NS’). In addition, a pre-existing non-coding mutation was fixed on segment 3 (marked ‘n’), while two *de novo* coding mutation appeared on segment 4 (marked ‘c’). The adapted (Ad) virus was generally very pure. Figure modified from (Bourret et al. 2013).

In a previous study (Bourret et al. 2013) we explored the ability of a duck influenza virus isolate of the H1 subtype to adapt to swine respiratory cells. We reported that *in vitro* passages in swine cells led to multiple genetic changes (Fig. 1). First, the original virus isolate was a mixture, i.e. had two different versions of 6 of the 8 gene segments. The adaptation to swine led to the fixation of a single version on all gene segments. Second, two *de novo* coding mutations were identified on segment 4 in the adapted strain. The present study goes beyond the simple description of gene frequency evolution provided in (Bourret et al. 2013), by investigating the functional impacts on viral fitness of changes on the different gene segments as well as their interactions. We also compiled a global, robust phylogeny where we mapped those individual mutations deemed to be the most consequential *in vitro*, highlighting their perceived role in the field. Finally, based on their predicted location in the 3D crystal structure of the hemagglutinin, we hypothesised that these mutations would influence virus membrane fusion during the entry process and confirmed this experimentally.

## 2 Materials and methods

### 2.1 Viruses and cells

The A/mallard/Netherlands/10-Nmkt/1999 (H1N1) duck influenza isolate (referred to as ‘Wt’, for wild-type) was passaged 10 consecutive times in Newborn Pig Trachea (NPTr) swine respiratory cells (Ferrari et al. 2003), obtained from APHA Weybridge, U.K. as described in Bourret et al. (2013). The swine cell-adapted stock was named ‘Ad’ (for ‘adapted’). Cell cultures were handled in an enhanced containment level 2 biosafety facility with restricted access and negative air pressure or in a containment level 3 biosafety facility as appropriate. Primary duck embryo fibroblasts (DEF) derived from embryonated duck eggs were maintained using Dulbecco's Modified Eagle Medium (DMEM) supplemented with penicillin (100 U/ml), streptomycin (50 µg/ml), amphotericin (2.6 µg/ml), l-glutamine (10 mM), sodium pyruvate (1 mM) and foetal calf serum (FCS; 10% v/v). Infections were carried out replacing FCS with 0.3% (w/v) bovine serum albumin (BSA) and adding 0.25 µg/ml trypsin (Worthington). Cells were cultured at 37 ºC in a 5% CO_2_ atmosphere.

### 2.2 Virus rescue

Virus rescues were carried out to retrieve the parental and adapted virus consensus as well as all 14 different reassortants, allowing us to perform growth rates assessment for all these variants. Virus rescue was conducted using the fusion-enhanced method described in Bourret et al. (2012). Briefly, this method involves adding a plasmid causing the expression of the Mædi-Visna virus envelope protein to a standard 8-plasmid rescue system. This causes cell-to-cell fusion, thereby increasing the chances of having all eight viral rescue plasmids expressed in a single syncytium and enhancing rescues of difficult strains.

### 2.3 Quantitative studies of viral growth properties

**(i) Laboratory protocols**. In order to study viral growth efficiency in culture, assay conditions were optimised to enable study of the viral growth rate during the exponential growth phase. Replicate infections were carried out in six-well plates seeded with 7.5 × 10^5^ cells per well and infected on the next day with 0.1 viral genome copies per cell. Cells were seeded in FCS-supplemented media and infected in FCS-free, trypsin-supplemented media. On the day of infection, the FCS-supplemented medium was removed, cells were washed with PBS and infected with 500 µl of infection medium containing 7.5 × 10^4^ viral genome copies per well. The inoculum was left onto the monolayer of cells for 1 h at 37 °C and 5% CO_2_ for the virus to adsorb. After 1 h, the inoculum was removed and the monolayer was rinsed with PBS to remove unadsorbed virus. The culture was then covered with 2 ml of fresh medium. Samples consisting of culture supernatant were taken at 1, 18, 24, and 30-h post-infection, time being counted from the moment the inoculum was put onto the cells. Samples were snap frozen at −70 °C and then processed for quantitation of viral yields. In each assay, uninfected wells were included as controls and handled in the same way as the tests wells; no evidence of viral growth was detected in these.

Genome copy numbers in the supernatants were estimated by real-time, quantitative PCR (qPCR). This approach was chosen because it allows an estimation of viral genome copy numbers that is independent from the infectivity of the virus in any given system, arguably making it the most objective quantitation of virus yield. Adaptation to different cell types may impact on apparent yield when assayed by cell culture methods, i.e. may affect the ‘efficiency of plating’, while the alternative of using HA titre is insufficiently sensitive to monitor virus yields below a certain threshold. The ratio of genome copies to plaque forming units (pfu) was very similar between the two rescued viruses rWt and rAd (2 × 10^3^ to 3 × 10^3^ copies-to-one-pfu for both viruses). A formal validation of the use of genome copy numbers to estimate exponential growth rate is provided in the Supplementary Methods.

The QiaAmp viral mini kit (Qiagen) was used for RNA extraction from allantoic fluids and cell culture supernatants (liquid samples) according to manufacturer’s instructions. Reverse transcription of viral RNA into complementary DNA (cDNA) was performed using Fermentas RevertAid Premium reverse transcriptase as per manufacturer’s instructions, using a primer specific for the 3' conserved region of the influenza A segment 7 viral RNA (vRNA) (MAFU: AGCAAAAGCAGGTAG; all primer sequences given from 5′ to 3′). Quantitative PCR (qPCR) was performed using Qiagen QuantiTect Probe PCR kit in 20 µl reactions containing 10 µl 2× PCR master mix, 0.4 µM of each primer (Sep1: AGATGAGTCTTCTAACCGAGGTCG, Sep2b: TGCAAAG ACATCTTCAAGTCTCTG), 0.2 µM of a dual-labelled probe (SePRO: 6FAM-TCAGGCCCCCTCAAAGCCGA-BHQ1a), 2 µl of template cDNA, and RNAse-free water to 20 µl. These primers and probe [named after (Löndt et al. 2008)] were modified from a set described in (Spackman et al. 2002) and were targeted at a conserved region of the M gene. Runs were performed on a Rotorgene 3000 machine (Corbett Research) and consisted of one initial activation step (95 °C for 15 min), followed by 45 cycles consisting of a denaturation step (94 °C for 15 s) and a combined elongation and acquisition step (60 °C for 60 s). A stock of quantitation standard was made from amplified and purified full-length M gene from virus Wt whose copy number was calculated using a PicoGreen (Invitrogen, used as per manufacturer's instructions) estimation of pure M gene DNA mass in the sample, the gene's molecular weight, and Avogadro's number. Standards were prepared fresh for every run by serial 10-fold dilutions of this stock. Standards behaved closely to theoretical expectations for this type of absolute quantitation. For most runs, efficiency of reaction ranged between 0.9 and 1.1, standard curve slope ranged between −3.2 and −3.5, detection cycle for a single copy of template ranged between 33 and 40, and fit between calculated standard curve and experimental standards (*R*^2^) was over 0.99.

**Figure 2.**
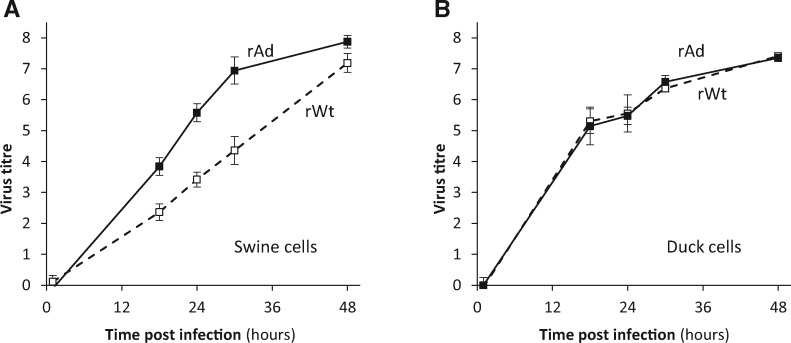
Comparison of the growth kinetics of virus rWt (open squares and dashed line) and rAd (filled squares and solid line) in swine (NPTr) and duck (DEF) cells. Virus titres are expressed as log(viral copies/µl supernatant). Shown at each time point is the mean±SD from three replicate infections.

**Figure 3.**
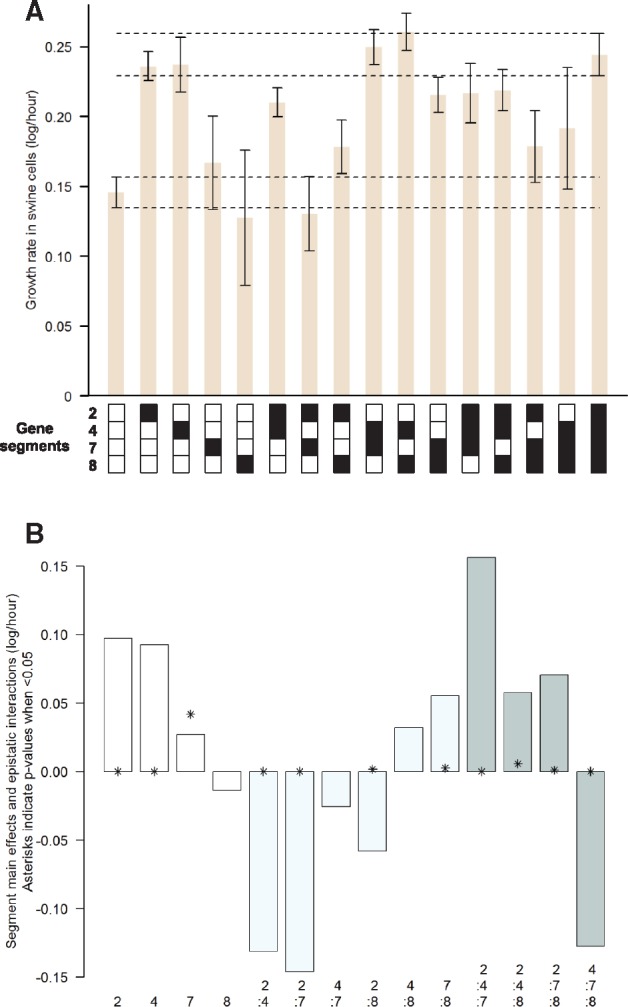
Fitness of reassortants and estimates of segment main effects and interactions in swine cells. (A) Estimated growth rate of all reassortant viruses in swine cells. The genetic makeup of the rescued viruses is represented below the histograms: the respective origin of segments 2, 4, 7, 8 is shown from top to bottom with white squares indicating segments from virus rWt and black squares indicating segments from virus rAd (i.e. mutated segments). Error bars show 95% confidence intervals derived from linear growth models. Dotted lines show the upper and lower limits of the 95% confidence intervals of viruses rWt (parental) and rAd (adapted). (B) Output from the minimal adequate linear model of growth rate as a function of the four segments, showing estimates of main effects (white bars) as well as two-way (light blue bars) and three-way (grey bars) interactions. For the statistically significant effects, asterisks indicate the *P* values (thus an asterisk closer to zero means a more significant effect). Raw data from the virus growth experiments are available as Supplementary File S1.

**Figure 4.**
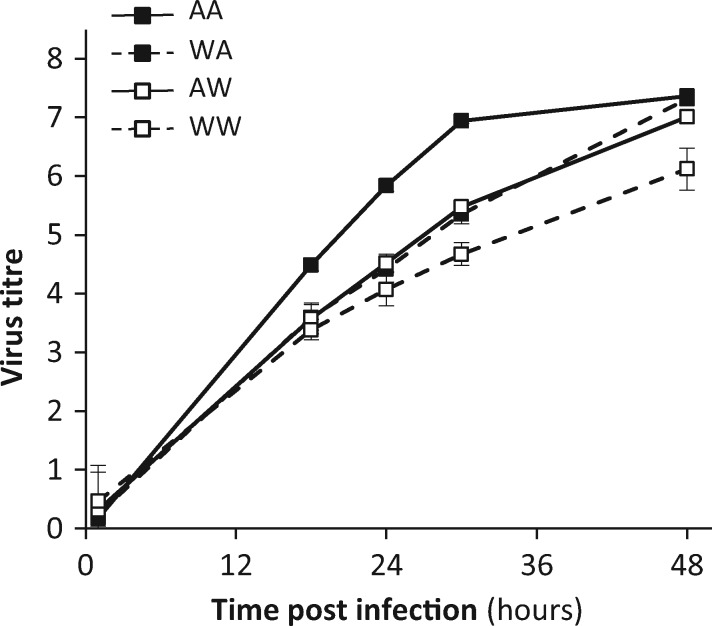
Growth kinetics of the WA and AW hemagglutinin single mutants (filled squares and dashed line, open squares and solid line, respectively) compared to those of a virus bearing the full parental (WW) or a double mutant (AA) hemagglutinin. Viral titres are expressed as log(viral copies/µl supernatant). Mean yield±SD from four replicate infections is shown for each virus at each time point.

**(ii) Mathematical modelling.** Viral yields from growth assays were log-transformed, and negative samples (i.e. no genome copy detected) were assigned a value of zero. The experimental protocol above was optimised so that growth curves between 1 h and 30 h post infection could be modelled by a straight line (as examples, see Figs 2 and 4). Exponential growth rates, expressed as log(genome copy number)/h, were estimated as the slope of these linear regressions (Fig. 3A), and this was the measure of fitness used in this study. Growth rate differences between different viruses were assessed by testing statistically the differences of the hour:virus interaction coefficients from the fitted minimum adequate mixed effect linear model (Table 1, top). A similar analysis considered the four gene segments as four explanatory factors (instead of virus identity as one factor) in order to assess segment main effects and epistatic interactions (Table 1, bottom). All statistical analyses were carried out in R (R Core Team 2014) and all codes are available upon request.

**Table 1.** Model selection to study (i) differences between the growth rate of different viruses (top) and (ii) epistatic interactions between viral gene segments (bottom). The starting maximal (a.k.a ‘beyond optimal’) models contained all interactions between fixed effects, as well as random effect structure estimated on both intercept and slope using lmer function in R (package lme4). They were simplified as recommended in (Crawley 2007; Zuur et al. 2009) to obtain a minimum adequate model usable for statistical analyses and interpretations.

Model objective	Maximal model	Minimum adequate model
Assess the effect of viruses	yield∼hour*virus + (hour|rep)	yield∼hour*virus-virus + (hour-1|rep)
	AIC: 340.70	AIC: 287.74
Assess the effect of viral gene segments	yield∼hour*seg2*seg4*seg7*seg8 + (hour|rep)	yield∼hour*seg2*seg4*seg7*seg8
	AIC: 340.70	-(seg2*seg4*seg7*seg8)
		*-hour:seg2:seg4:seg7:seg8*
		* + (hour-1|rep)*
		AIC: 283.94

*yield:* log_10_ of viral copies/ml supernatant; *hour*: hours post infection; *virus*: identity of viral construct; *seg2*: allele for segment 2 (‘Wt’ or ‘Ad’), likewise for seg4, seg7 and seg8; *rep*: identity of replicate infection, as a random effect.

*AIC*: Akaike’s Information Criterion, calculated as -2*log-likelihood + 2*n_parameters_.

### 2.4 Erythrocyte lysis assays for a range of pH

Hemagglutinin-mediated red blood cell membrane lysis activity was assessed for the various hemagglutinins of interest in an A/Puerto Rico/8/34 backbone in wells containing 100 µl of a 1% (v/v in PBS) chicken red blood cell (RBC) solution and 64 hemagglutination units of virus in 50 µl. Chicken RBC were purchased from TCS Biosciences (#FB010AP). The A/PR/8/34 backbone was used to segregate HA-mediated effects from those of the neuraminidase (which was highly active for the rescued avian viruses and interfered with the performance of the fusion assay by causing intense virus elution). After a 30-min hemagglutination time, 150 μl of citric acid buffer at varying pH (4.0–6.0 range) was added to each well and the plate was incubated for 30 min at 37 °C. The plate was centrifuged at 2000 rpm for 8 min at 4 °C and 150 μl of supernatants from each well were transferred into a 96-well, flat-bottomed plate. Hemoglobin release was quantified by measuring absorption at 450 nm using a Biorad iMarc Microplate Reader. A negative control was included at each pH with no virus added. A 100% lysis control was obtained using 0.2% Triton X-100 (170 µl of PBS, 30 µl 2% Triton X-100, 100 µl RBCs).

### 2.5 Allele distribution in the field and viral phylogenetic analyses

Influenza A H1 hemagglutinin nucleotide sequences were downloaded from the NCBI Influenza resource (Bao et al. 2008). Queries included all type A influenza from avian or swine hosts, any country/region, and any neuraminidase subtype, including pandemic H1N1 viruses, lineage defining strains, the FLU project, vaccine strains, and mixed subtypes, but excluding ‘laboratory’ strains. This resulted in a query of 7404 sequences, as of 2015-05-14. Thirteen sequences with more than seven fully ambiguous (N) nucleotides were excluded as these may represent poor sequence quality. A further 2408 sequences were excluded on the basis that they were less than 1500 base pairs long; many of these were missing HA2, the region of interest. The longest open reading frame was extracted and translated using getorf and transeq from the EMBOSS software suite v.6.6.0 (Rice et al. 2000), and amino acid sequences were aligned using MUSCLE v.3.8.1551 (Edgar 2004). Nucleotide sequences were mapped to the amino acid sequences using pal2nal v.14 (Suyama et al. 2006), and trimmed automatically using trimA1 v1.4 (Capella-Gutiérrez et al. 2009), omitting sites with gaps in more than 20% of the sequences, resulting in 4985 sequences. We also processed the results table from NCBI, to classify hosts as swine, aquatic birds, terrestrial birds, or other. We retained sequences from swine and aquatic birds, and excluded sequences for which country or date information were missing, resulting in a dataset of 4942 sequences; 961 of these sequences were identical to others, and so were excluded from phylogenetic analysis, resulting in a dataset of 3981 sequences, 3520 from swine, and 461 from aquatic birds.

A maximum likelihood tree was reconstructed using ExaML v. 3.0.11 (Kozlov et al. 2015) using a general time reversible model of nucleotide substitution, and rate variation modeled using a position-specific model with 25 rate categories. This tree demonstrated three major swine influenza clades, one of which was derived from the aquatic bird influenza pool. We therefore focused subsequent analyses on the clade consisting of the aquatic bird gene pool and the swine sequences derived from it (*n* = 846 sequences, 458 from aquatic birds and 388 from swine). We reconstructed changes at position 67 and 113 in HA2 (see Results) by parsimony. We confirmed that the S113F transition occurred coincident with the jump to swine using BEAST v.1.8.2 (Drummond et al. 2012). We assumed an SRD model of substitution (Shapiro et al. 2006) for the nucleotide sequences, and discrete trait models (allowing asymmetric rates) for host type, continent, and amino acid variants at positions 67 and 113. A strict molecular clock was assumed for all changes, and the effective population size was assumed to be constant. Tipdates for the sequences accommodated variation in the precision that collection dates were recorded (year, year and month, or full date). Markov Chain Monte Carlo was run for 250 million iterations, including a burn-in of 100 million iterations. A single maximum clade credibility tree was generated using TreeAnnotator, part of the BEAST package. Most of the swine sequences belonged to a single clade. We extracted the sequences for this clade (*n* = 357 unique amino acid sequences), and tested for further diversification following the host jump using FADE, an approximate but fast approach to screening amino acid sites (and variants at those sites) for directional selection, as implemented in Datamonkey (Delport et al. 2010). We assumed an influenza HA-specific model of amino acid substitution for FADE, and the rooted subtree obtained from the Bayesian MCC tree. Trees were visualised, coloured and annotated using FigTree v1.4.2 (http://tree.bio.ed.ac.uk/software/figtree/). Assignment of swine H1 hemagglutinins to their respective clade (avian-like, classical, triple reassortant, pandemic 2009, and other human-derived) was based on a number of published studies (Brown et al. 1998; Marozin et al. 2002; Karasin et al. 2006; Smith et al. 2009a, 2009b; Vijaykrishna et al. 2010; Vijaykrishna et al. 2011; Lange et al. 2014; Ozawa et al. 2015).

## 3 Results

To investigate the molecular pathways of adaptation of a duck influenza A virus to swine respiratory cells, we assessed the outcome of passaging influenza A/mallard/Netherlands/10-Nmkt/1999 (H1N1) in NPTr cells.

The parental virus stock is hereafter referred to as ‘Wt’ (for ‘wild type’) and the swine cell-passaged stock as ‘Ad’ (for ‘adapted’), and in-depth genome analyses of both viral stocks have been described in detail previously (Bourret et al. 2013).

### 3.1 Reverse genetics studies of reassortant viruses

Figure 2 shows the comparison of the growth kinetics of a rescued virus bearing all eight consensus segments from the parental virus Wt (hereafter rWt) and a rescued virus bearing all eight segments from the adapted virus Ad (hereafter rAd). In NPTr cells, rAd showed a clear growth advantage over rWt with exponential growth rates of 0.244 log/h for rAd and 0.146 log/h for rWt. This means that in the exponential growth phase, rAd would achieve one extra log_10_ difference in yield over rWt every 10 h (*P* < 0.001). A similar growth rate assessment in duck embryo fibroblasts (DEF) showed no difference between the two viruses in duck cells (Fig. 2B;*P* = 0.943), suggesting that the effect on growth rate was cell-type dependent.

The rWt and rAd viruses differed by a total of 25 amino acids distributed on six described viral proteins as follows (number of differing amino acids between square brackets): PB1 [7], PB1-F2 [11], HA [2], M42 [2], NS1 [2], NS2 [1]. An additional point mutation on segment 3 also became dominant during the experimental adaptation process, but was not studied further here as it did not modify any known influenza protein. As the selected proteins are encoded by gene segments 2, 4, 7, and 8, we sought to investigate the respective contribution of these four gene segments using reverse genetics in the common backbone of segments 1, 3, 5, and 6 (Fig. 3A). Our full-factorial design enabled testing the individual effects of each segment as well as their interactions. Modelling viral growth as a function of gene segment identity (Fig. 3B) indicated that segments 2 and 4 from the adapted virus have major positive main effects on virus growth. In contrast, segment 7 has only a weak (but significant) positive main effect and segment 8 has no main effect. Interestingly, we found strong epistatic interactions between these segments. The magnitude and the sign of these interactions varied substantially between segment combinations, while their global average did not significantly depart from zero (−0.011, *P* = 0.726). The two-way epistatic interactions tended to be more negative than the three-way interactions (average two-way epistasis = −0.045 versus three-way epistasis = 0.039, *P* = 0.217). The two-way epistasis between the three pairs of segments with positive main effects was even more negative (−0.100, *P* = 0.116) although none of these average values were statistically different from zero. However, the epistatic interaction between the two most beneficial segments (2 and 4) was strongly and very significantly negative (−0.131, *P* < 0.001). In fact, we found some evidence for sign and double sign epistasis as the 2 + 4 double mutant had lower fitness than both the virus carrying only the mutated segment 2 (*P* = 0.024) and the virus carrying only the mutated segment 4 (*P* = 0.054) (Fig. 3A and B). Similarly, the epistasis between segments 2 and 7 was also very strongly negative (−0.146, *P* < 0.001), and the 2 + 7 ‘double mutant’ had lower fitness than both the virus carrying only the mutated segment 2 (*P* < 0.001) and the virus carrying only the mutated segment 7 (*P* = 0.002).

The significant main effects of segments 2 and 4 indicated that the specific mutations carried by these segments were involved in adaptation to swine respiratory cells. Segment 2 of the adapted virus carried 18 coding and 96 non-coding mutations while segment 4 carried only 2 mutations, both of them coding. Moreover, all of the nucleotide differences observed in segment 2 originated simultaneously during the adaptation due to the selection of a reassortant from a low-level subpopulation in the initial virus inoculum (Bourret et al. 2013). Thus, any number or combination of these changes might be responsible for the phenotype. In contrast, the two mutations in segment 4 appeared *de novo* during the adaptation experiment. It was thus anticipated that these two segment 4 changes could each be very consequential, and in the following we focus on these two mutations to explore in more details their effect on viral growth.

### 3.2 Impact of single hemagglutinin mutations on viral growth rate

In order to determine the individual contribution of each these two hemagglutinin changes, we rescued the two chimeric constructs each bearing a single mutation in an otherwise rWt backbone. The two mutations in question are G67S and S113F on the HA2 subunit, numbering from the N-terminus of the fusion peptide (equivalent to G411S and S457F of the full-length HA0). The Wt and Ad hemagglutinin alleles are designated ‘WW’ and ‘AA’ respectively and the two single mutants are ‘WA’ and ‘AW’. Figure 4 shows that the two single mutants had a comparable growth phenotype, that was intermediate between that of rWt and that of a construct bearing Ad’s segment 4 in a rWt backbone.

**Figure 5.**
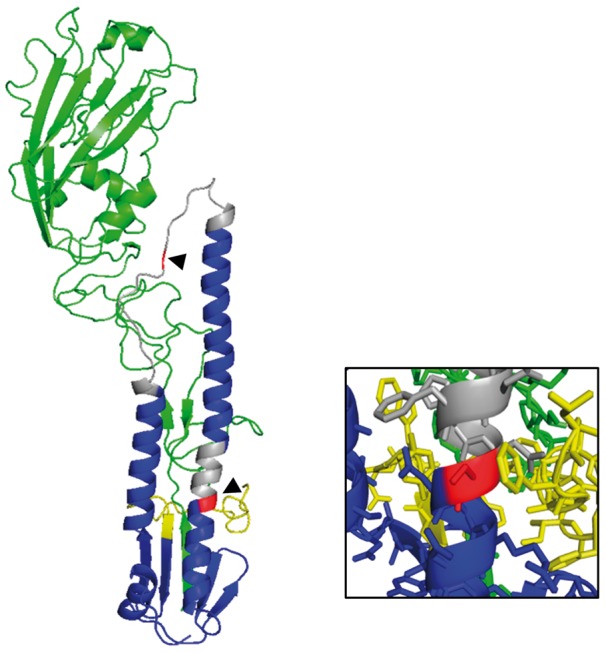
Schematic illustration of the situation of the two mutations reported here within the H1 hemagglutinin structure for wild duck strain WDK/JX/12416/2005. The mutated positions are shown in red and indicated by arrowheads. Green indicates the HA1 subunit, blue indicates the HA2 subunit with the fusion peptide in yellow and regions changing conformation at acidic pH to trigger fusion in grey. The insert shows a close up of the HA2 residue 113 region with side chains displayed (the wild-type serine 113 is shown). Residue 113 is close to a phenylalanine side chain at position 3 of the fusion peptide. The structure was generated based on Protein Data Bank file accession number 3HTO (Lin et al. 2009) using the PyMOL molecular graphics system version 1.7.5.0.

The HA2 G67S mutation is located in the middle of a 22-aminoacid region that undergoes a conformational change from a loop to an α-helix at acidic pH, contributing to fusion of the viral and endosomal envelopes (Fig. 5). The HA2 S113F mutation is located at the C-limit of an α-helix just adjacent to a six-aminoacid region which changes from α-helix to loop at acidic pH, also contributing to fusion (Sriwilaijaroen and Suzuki 2012). Mutation from the small hydrophilic serine (Fig. 5, insert) to the large hydrophobic phenylalanine at that position might alter the interaction of this region with the fusion peptide in the neutral pH form of the HA trimer. Notably there is clear potential for an interaction with the phenylalanine located at position 3 of HA2. Since acid-triggered fusion of the viral and endosomal envelopes is a vital step for the viral replication cycle to proceed, we investigated whether these mutations impacted the envelope fusion process, and notably its sensitivity to acid triggering.

### 3.3 Effect of the hemagglutinin mutations on fusion pH

**Figure 6.**
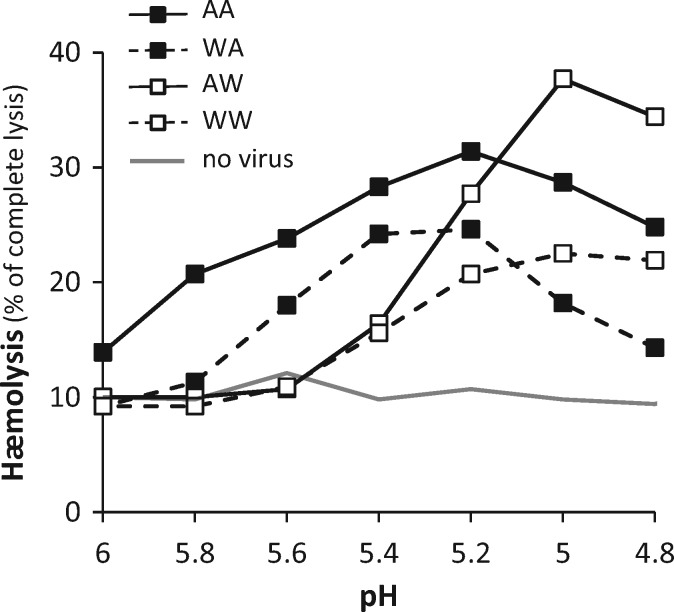
Impact of the two segment 4 mutations on pH-dependent, hemagglutinin-mediated membrane fusion. Four viral constructs were tested for their ability to induce fusion-mediated hemolysis when exposed to various acidic pH.

To test the effects of the mutations on the membrane fusion process, the four possible combinations of alleles were tested in a quantitative hemolysis assay where excess virus bound to RBC membranes results in release of hemoglobin upon low pH exposure. Red blood cell lysis was dependent upon the presence of virus in the pH 4.8-6 range, each of the 4 viruses showing a distinct pH profile with a characteristic threshold pH and optimum pH for lysis (Fig. 6). The Wt hemagglutinin (WW) showed optimum lysis at pH 5 and a threshold for lysis of pH 5.4. The single mutants revealed that the G67S mutation (AW) resulted in a marked increase in lysis efficiency without altering the threshold or optimum pHs. In contrast the S113F mutation (WA) had little effect on lysis efficiency but resulted in an increase in the threshold and optimum pH (5.6 and ∼5.2, respectively). The double mutant (AA) showed greater lysis efficiency, optimum lysis at pH 5.2 and a threshold of pH 5.8 (with some evidence of lysis even at pH 6). It is also notable that lysis fell markedly for WA (and to a lesser extent AA) when the pH was 4.8–5.0. Such bell shaped curves have been reported before for other strains of influenza virus (Maeda and Ohnishi 1980; Huang et al. 1981), and suggest that under conditions beyond the optimal pH, triggering of the HA conformational change results in less efficient membrane lysis.

### 3.4 Occurrence of HA2 G67S and S113F in natural field isolates

**Figure 7.**
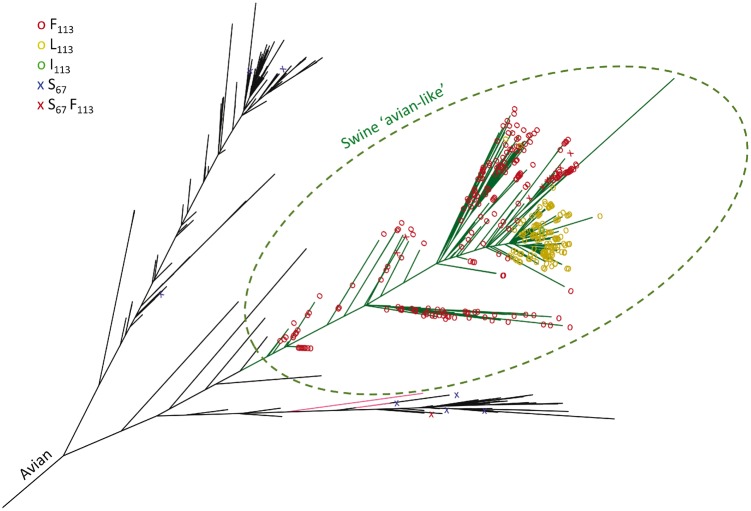
Evolutionary relationships among H1 hemagglutinins of influenza A viruses from water birds (black branches) and the avian-like swine influenza clade (green branches; pink branches denote other swine strains that do not form part of the ‘avian-like’ swine influenza clade). A dark red marker indicates a phenylalanine at HA2 position 113, a yellow marker indicates a leucine and a bright green marker indicates an isoleucine. Serine at HA2 position 67 is indicated by a cross (blue if associated with serine at position 113, dark red if associated with a phenylalanine at position 113).

The importance *in vitro* of the two hemagglutinin mutations identified in this study for viral fitness in swine tracheal cells (Figs 3 and 4) led us to assess their relevance in the field. To this end, we first compared the distribution of these alleles in waterfowl *versus* swine influenza isolates. We reconstructed a global maximum-likelihood phylogeny of swine and aquatic birds H1 hemagglutinins of 3981 sequences (3520 from swine and 461 from aquatic birds), and mapped the HA2 mutations identified in this report onto the phylogeny. The phylogeny showed three major swine influenza clades (Supplementary Figs S2 and S3), one of which results clearly from a host jump from the aquatic bird influenza pool (Fig. 7). We therefore focused analyses on the clade consisting of the aquatic bird gene pool and the swine clade derived from it (i.e. 846 sequences). Strikingly, the vast majority (close to 98%) of these swine strains, which form the so-called ‘avian-like’ swine influenza clade, were mutated with a phenylalanine (dark red marker) or leucine (yellow marker) at position 113 compared to the ancestral avian consensus (Fig. 7). By contrast, mutation at position 113 has not been recorded so far in aquatic bird viruses in the field (its apparent occurrence in the duck clade on Fig. 7 results from the presence of virus ‘Ad’ in the databases).

We reconstructed ancestral changes at position 67 and 113 in HA2 by parsimony, and confirmed that the S113F transition had occurred coincident with the jump to swine using BEAST (Drummond et al. 2012). A few members of the swine clade also carried the G67S allele, *i.e.* had the AA genotype of rAd (dark red crosses), while a few waterfowl isolates had a serine at position 67 (crosses). We also used the FADE method to test whether there was statistical evidence for ongoing diversification at the amino acid level as might be anticipated for adaptational “fine-tuning” observed for previous cross-species transitions (Bhatt et al 2013). Significant directional selection (posterior probability of 0.99 or greater) was detected at 43 sites out of 565, including HA2 position 113 with directional selection towards phenylalanine in the swine clade relative to the rest of the alignment. Following the host jump, there was also evidence for mutation towards leucine. The emergence of that clade in swine was estimated to have occurred about 42 years ago with the most ancient field strains obtained for that clade dating back some 37 years (Pensaert et al. 1981). A single maximum clade credibility tree (in the .tre format) showing the H1 hemagglutinins of the aquatic bird influenza gene pool and the swine influenza sequences derived from it, including all ancestral reconstructions, statistical data and molecular clock results, is available for download as supplementary online material.

The two other major swine clades, which do not appear to be recently nor directly derived from the aquatic bird reservoir (the ‘classical’ swine clade with its descendants and a third clade composed of other human-derived swine hemagglutinins) showed rarer, sporadic occurrences of 113F or 67S (Supplementary Figs S2 and S3).

## 4 Discussion

The objective of this research was to study the effects of mutations involved in the adaptation of an H1 avian influenza virus to a swine host. Experimental adaptation to swine cells started from a polymorphic uncloned, low egg-passage avian field-isolate population consisting of a mixture of viruses (Bourret et al. 2013) representative of the natural diversity present at the inception of a cross-species transmission event. Since the ability to replicate quickly is a crucial factor in the ultimate success or failure of an infection confronted with an active host immune response (Ehrhardt et al. 2010; Crisci et al. 2013), we measured adaptation as an increase in growth rate. Faster growth in swine tracheal cells was observed as a result of experimental evolution of a duck strain in these cells. This is not surprising as it is expected that variants with a growth speed advantage will be selected upon serial passaging in a given system. Unlike in NPTr however, there was no difference in growth kinetics between rWt and rAd in duck embryo fibroblasts (DEF). This is consistent with the adaptation overcoming a deficiency or impediment in the virus’ interaction with the NPTr cells, rather than gaining a general growth advantage.

### 4.1 Genetics of virus adaptation

Experimental evolution led to the loss or fixation of minority alleles through reassortment and the fixation of two *de novo* spontaneous mutations. Reverse genetic studies of all reassortants allowed us to explore the individual effects of the preferentially selected segments of the adapted virus as well as the interaction between these segments. Two segments (2 and 4) stood out in this analysis as being associated with important main effects on virus growth. In particular a clear overall growth advantage was mapped to two novel mutations on segment 4 encoding the hemagglutinin. In addition, our full-factorial design demonstrates the existence of significant epistatic interactions among segments. These epistatic interactions, including sign epistasis, suggest that the fitness landscape may be characterized by several fitness peaks that may be attained by different combinations of segments. Interestingly, the two-way epistatic interactions between adaptive mutations tended to be negative. This conforms to expectations in a single peak fitness landscape where the convex shape of the peak implies that the fitness gain associated with each beneficial mutation decreases when the phenotype is closer to the optimum (Martin et al. 2007). Further studies with greater statistical power are warranted to investigate other patterns such as potential systematic differences between two-way and higher order interactions. It would also be interesting to study the fitness of these different reassortants in the original environment, where the described epistatic interactions could further inform discussions of the potential costs in the original host associated with adaptation to a new host (Lalić and Elena 2013).

### 4.2 Host jump in the field

We showed the relevance of the mutation at HA2 position 113 in the field with a global maximum likelihood phylogenetic reconstruction. This analysis confirmed that while all field aquatic bird isolates surveyed to date have a serine at that position, the vast majority of swine isolates from their most successful and consequential descendent swine clade, i.e. the ‘avian-like’ swine clade, have a phenylalanine or leucine. The host jump itself was found to be associated with a mutation from serine to phenylalanine, identical to that observed experimentally in this study. This suggests that replacement of a small, polar amino acid (serine) with a large, hydrophobic residue such as phenylalanine or leucine at HA2 position 113 may be an important step in the direct adaptation of fully avian influenza viruses to swine. At HA2 position 67, the species association pattern was less clear, with the mutated amino acid (serine) being infrequent in all clades. This further implies the existence of strong epistatic interactions in this virus, as there may be other mutations that can substitute for 67S in helping potentiate the role of 113F(L) in the successful establishment of a swine influenza lineage from aquatic bird precursors.

### 4.3 Mechanism of action of the HA mutations

Hemagglutinin-mediated, pH-dependent membrane fusion is an essential process in the viral replication cycle. Following endocytosis by the cell, acidification of the endosome triggers an irreversible change in HA conformation that causes the viral envelope to fuse with the endosome membrane. This allows the viral genetic material to enter the cytoplasm, paving the way for all subsequent steps of the replicative cycle. Here, pH dependent lysis patterns showed that the two HA mutations had distinct phenotypic effects on the fusion process. Fusion of the ‘WA’ single mutant is likely to occur at a higher pH whereas for the ‘AW’ single mutant the process occurs more efficiently. The two mutations operate cooperatively, to induce greater fusion at higher pH threshold, and these effects were well correlated with the growth speed phenotypes brought about by the two hemagglutinin mutations alone and in combination in swine cells (Fig. 4). The increase in fusion pH and efficiency may enable earlier escape from the endosome during its acidification process. Although some have hypothesized that early escape might be detrimental to the virus as it may be more readily sensed by cytoplasmic RNA pattern recognition receptors (reviewed in Mair et al. 2014), we show here that earlier and more complete fusion correlates with better growth in swine cells. There are various, non-mutually exclusive mechanisms that can be proposed to account for this. First, earlier escape from the endosome could simply shorten the duration of the replicative cycle. Alternatively, early escape might avoid exposure to innate immunity factors such as the interferon-induced transmembrane protein IFITM3 (Brass et al. 2009), which acts at the later stages of endocytosis to prevent the cytoplasmic entry of viral genomes (Feeley et al. 2011). Whether or not either of these hypotheses is correct, an explanation for why growth enhancement is not observed in DEFs is warranted. Perhaps the endosomal pH profile in duck cells is different, or the duck-derived Wt virus is not susceptible to duck IFITM. Further investigation into the potential role of IFITM in host tropism would include studying whether the mutant virus retains its growth advantage in swine cells differentially expressing IFITM.

A higher fusion pH threshold would result in reduced infectivity after exposure to an acidic environment (i.e. reduced acid stability) (Reed et al. 2010; Krenn et al. 2011; Koçer et al. 2013; Zaraket et al. 2013; Baumann et al. 2016) and the absence of 113F in duck field viruses could reflect a greater need for acid stability in duck compared to swine transmission cycles (either within the host or the conditions of their environment). Transmission is mainly fecal-oral in ducks, with the replication cycles chiefly happening in the lower intestine (Webster et al. 1978). Virus presumably has to survive transit through the proventriculus and gizzard, which are hostile low pH environments (∼pH 3 and pH 2, respectively) and acid stability is expected to be important (as also independently proposed by others (Baumann et al. 2016)). Mutations decreasing acid stability could therefore be selected against in the natural duck transmission cycles, consistent with the rarity of 113F in these hosts. A study of mutations altering the fusion pH of an H5 high pathogenicity avian influenza virus (HPAI) inoculated in ducks corroborates this hypothesis (Reed et al. 2010).

Other studies on this topic have been conducted in laboratory animal models (Krenn et al. 2011; Koçer et al. 2013), that are consistent with ours in suggesting that the optimal fusion pH for *in vivo* virulence in mammals is ∼5.2–5.4. Conversely, greater acid stability has been demonstrated for a number of duck compared to human viruses (Webster et al. 1978), although this trend is not absolute (Galloway et al. 2013) and we are probably only beginning to unravel the complexities of the involvement of fusion pH in influenza host range (Mair et al. 2014). Of note, mutations elevating or lowering the fusion pH are not limited to HA2 and several have been detected in the HA1 component (Keleta et al. 2008; Reed et al. 2010).

This study of the genetics of adaptation of H1 influenza to swine respiratory cells identifies mutations that may be involved in faster growth in this novel environment. Epistatic interactions on fitness suggest that the fitness landscape of influenza is complex and several combinations of mutations may lead to successful transmission to swine. Nonetheless the mutation at HA2 position 113 stands out in this analysis. Future studies should be carried out to help characterize other consequences of this mutation *in vivo*. Yet the phylogenetic analysis carried out in this study clearly demonstrates the relevance of these mutations in the field. Influenza virus surveillance in swine should thus consider S113F/L as a signature mutation indicative of a potential incipient emergence event.

## Supplementary Material

Supplementary Data
