# Supplementary material for: Adaptation of avian influenza virus to a swine host
Source: Virus Evol. 2017 Mar 18;3(1):vex007. doi: 10.1093/ve/vex007 (PMC5399929; doi:10.1093/ve/vex007)
Supplement: Supplementary Data [file vex007_Supp.zip › Caption to supplementary files.pdf]

Caption to supplementary files:

Supplementary Methods file.

Supplementary file S1 *(.csv file).* Titres from the virus growth experiments. Titres are expressed as $\log_{10}$(genome copies/µL supernatant) as a function of viral genetic makeup over the exponential growth phase, *i.e.* 1h-30h post infection.

Supplementary figure S2. Evolutionary relationships among all H1 hæmagglutinins from water birds and swine inferred using maximum likelihood (ML). Viral lineages are indicated by branch colours as follows. Black: avian. Green: avian-like swine. Blue: classical swine. Purple: swine triple reassortant. Orange: 2009 pandemic lineage. Brown: other human-derived swine H1 lineages. A dark red marker indicates a phenylalanine at HA2 position 113, a yellow marker indicates a leucine and a bright green marker indicates an isoleucine. Serine at HA2 position 67 is indicated by a cross (blue if associated with serine at position 113, dark red if associated with a phenylalanine at position 113). Scale bar at bottom indicates 0.05 substitutions per site.

Supplementary figure S3. Full resolution ML phylogram detailing all viruses used in the analysis with GenBank accession number, host, continent, year of sampling, subtype, and amino acids at positions 67 and 113. Branch colours indicate lineages as in figures 7 and S4, and tip labels are colour-coded according to position 113 (blue for serine; dark red for phenylalanine; yellow for leucine; bright green for isoleucine). Scale bar at bottom indicates 0.05 substitutions per site.

Supplementary file S4 *(.tre file).* Single maximum clade credibility tree showing the H1 hæmagglutinins of the aquatic bird influenza gene pool and the swine influenza sequences derived from it, including all ancestral reconstructions, statistical data and molecular clock results. The file can be opened and all information can be visualised using the figtree software, freely available at http://tree.bio.ed.ac.uk/software/figtree/
