# Supplementary material for: Adaptation of avian influenza virus to a swine host
Source: Virus Evol. 2017 Mar 18;3(1):vex007. doi: 10.1093/ve/vex007 (PMC5399929; doi:10.1093/ve/vex007)
Supplement: Supplementary Data [file vex007_Supp.zip › Supplementary Methods.pdf]

Supplementary Methods

to

Adaptation of avian influenza virus to a swine host

by

Vincent Bourret, Jon Lyall, Simon D. W. Frost, Angélique Teillaud, Catherine Smith, Sarah Leclaire, JinQi Fu, Sylvain Gandon, Jean-Luc Guérin, Laurence Tiley

***

On the validity of using genome copy numbers to estimate viral growth rates during the exponential growth phase

To confirm the validity of our experimental approach to estimate growth rate using genome copy numbers, we analyse a simple epidemic model that describes the initial growth of a virus population *in vitro*. In this model we assume that the resource (*i.e.* the number of susceptible host cells) is unlimited and virus growth is density independent. This is the case during the initial phase of our *in vitro* assay where the growth remains exponential during the first 30 h p.i. (see figures 2 and 4, main text).

We track the density of infectious - y_t- and defective - z_t- virus particles across time t. Initially a quantity y_0 and z_0 of infectious and defective virus, respectively, is introduced. The infectious particles replicate with a rate r, and we assume that a fraction d of the virus produced are defective. This yields the following dynamical system (the dot refers to differentiation with time):

$$\dot{y}_t = r(1-d)y_t$$
$$\dot{z}_t = dy_t$$

Solving these equations yields:

$$y_t = y_0 e^{r(1-d)t}$$
$$z_t = y_0 \frac{d}{r(1-d)}\left(e^{r(1-d)t} - 1\right) + z_0$$

The exponential growth rate of the density of the population of infectious viruses is: $r(1-d)$. This is the quantity we want to estimate for the different virus strains (*i.e.* the growth rates presented on figure 3A of the main text).

However, our assay does not measure the density of infectious virus but the *total density* of virus which is:

$$n_t = y_t + z_t = y_0\left(e^{r(1-d)t} + \frac{d}{r(1-d)}\left(e^{r(1-d)t} - 1\right)\right) + z_0$$

As expected, the density of the total population is higher than the density of infectious particles. After a transitory phase the relative proportion of infectious and defective particles becomes stable and both y_t and n_t have the same exponential growth rate: $r(1-d)$.

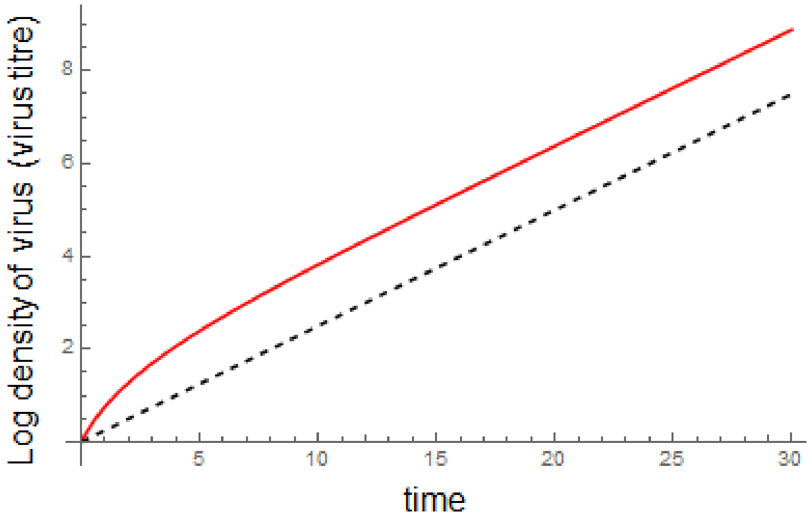

The above figure plots the log of y_t(dashed black) and the log of n_t(red) for the following parameter values: $y_0 = 1, z_0 = 0, r = 1, d = 0.75$. In this example, it is assumed that only infectious particles were inoculated (i.e. $z_0 = 0$). Note that after a transitory phase the slopes of the two lines are identical.

Our experimental procedure estimates the slope of the change in the log density of the total number of virus (using 4 time points: 1 h, 18 h, 24 h, 30 h and 3 replicates for each virus strain). This is also an estimate of the exponential growth rate of the population of infectious virus and thus provides a measure of the replication ability of the virus *in vitro* (the growth rate presented on figure 3A).
