# Supplementary figures and images for: Adaptation of avian influenza virus to a swine host
Source: Virus Evol. 2017 Mar 18;3(1):vex007. doi: 10.1093/ve/vex007 (PMC5399929; doi:10.1093/ve/vex007)

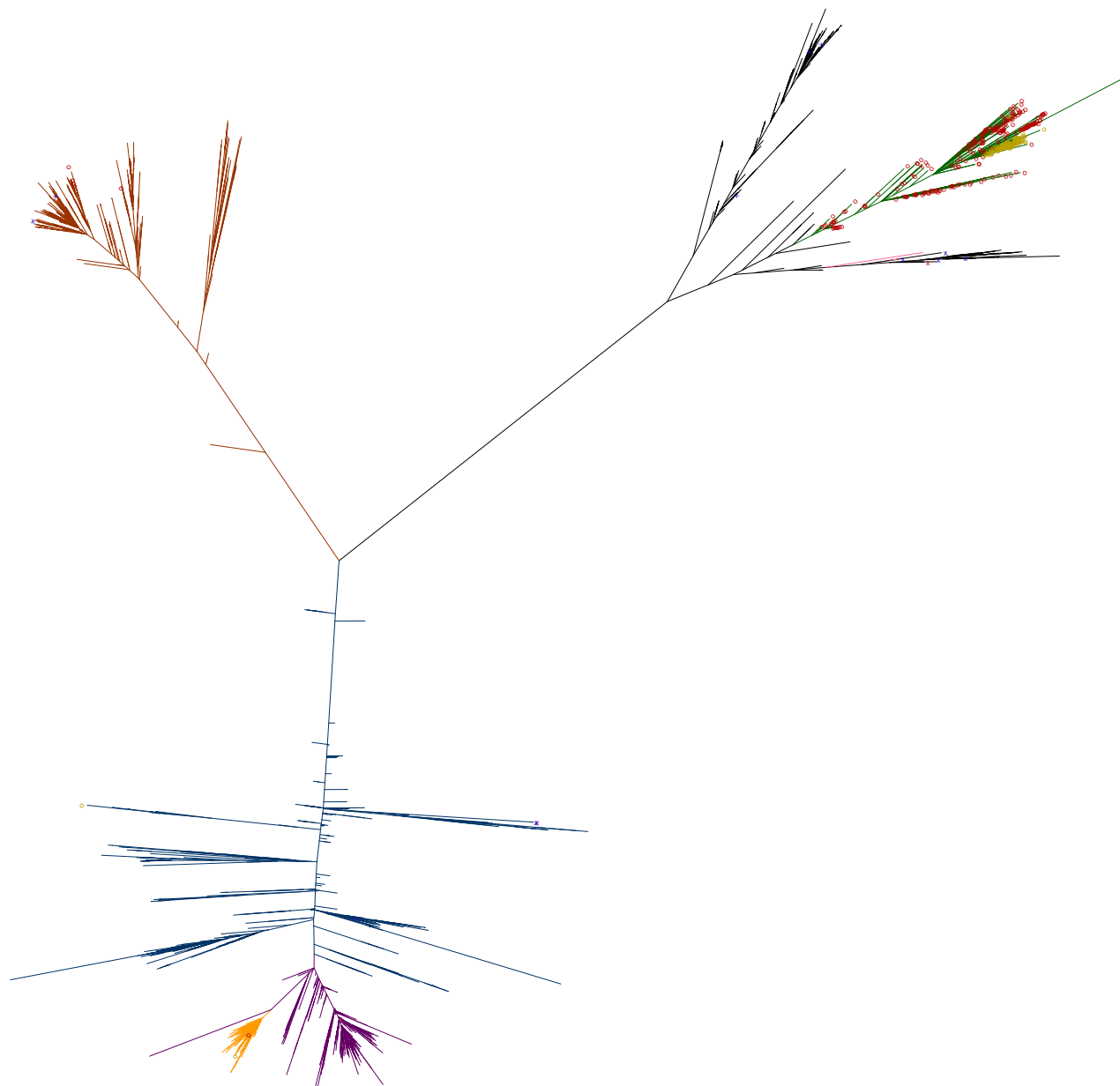

Supplement: Supplementary Data [file vex007_Supp.zip › S2 smaller.pdf]

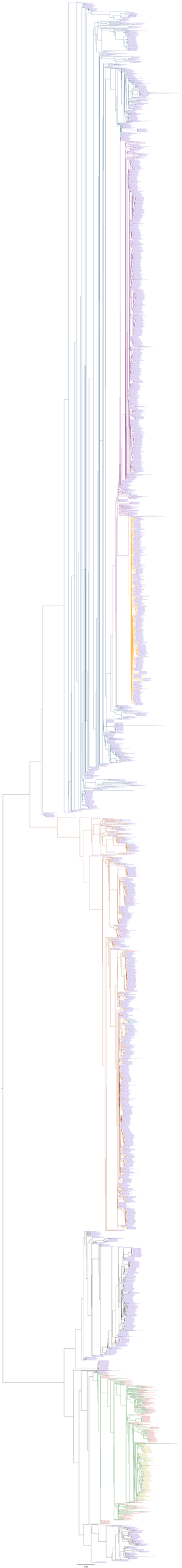

Supplement: Supplementary Data [file vex007_Supp.zip › S3 smaller.pdf]
